# Determinants of Quality of Work Life among Nurses Working in Hawassa Town Public Health Facilities, South Ethiopia: A Cross-Sectional Study

**DOI:** 10.1155/2017/5181676

**Published:** 2017-12-12

**Authors:** Lolemo Kelbiso, Admasu Belay, Mirkuzie Woldie

**Affiliations:** ^1^Department of Nursing, Wolaita Sodo University, Wolaita Sodo, Ethiopia; ^2^Department of Nursing, Jimma University, Jimma, Ethiopia; ^3^Department of Health Policy and Management, Jimma University, Jimma, Ethiopia

## Abstract

**Background:**

A high quality of work life (QWL) is a crucial issue for health care facilities to have qualified, dedicated, and inspired employees. Among different specialties in health care settings, nurses have a major share among other health care providers. So, they should experience a better QWL to deliver high-quality holistic care to those who need help.

**Objective:**

To assess the level of quality of work life and its predictors among nurses working in Hawassa town public health facilities, South Ethiopia.

**Methods:**

A facility based cross-sectional study was conducted on 253 nurses of two hospitals and nine health centers. The total sample size was allocated to each facility based on the number of nurses in each facility. Data were collected using a structured questionnaire. The interitem consistency of the scale used to measure QWL had Cronbach's alpha value of 0.86. A multinomial logistic regression model was fitted to identify significant predictors of quality of work life using SPSS version 20.

**Results:**

The study showed that 67.2% of the nurses were dissatisfied with the quality of their work life. We found that educational status, monthly income, working unit, and work environment were strong predictors of quality of work life among nurses (*p* < 0.05).

**Conclusion:**

Significant proportions of the nurses were dissatisfied with the quality of their work life. The findings in this study and studies reported from elsewhere pinpoint that perception of nurses about the quality of their work life can be modified if health care managers are considerate of the key issues surrounding QWL.

## 1. Introduction

The quality of work life (QWL) is a process by which the organizations' employees and stakeholders get an insight into how to work better together to improve both the staff's quality of life and the organizational effectiveness simultaneously. This concept basically pronounces the way by which an organization can safeguard the holistic well-being of an employee rather than only concentrating on job-related features [1].

QWL is a multidimensional idea which describes an employee's emotion regarding several aspects with respect to work. These include the job content, working situations, fair and adequate compensation, career advancement chances, duty discretion, involvement in decision making, occupational health and safety, work stress, employment security, organizational and personal relations, and work life stability [2–5].

QWL is usually supposed to be one of the most important elements in staffing and retaining, which has a great impact on holding the required number of nurses in each health care facility. To deal with the problem, the range of issues includes workload, professional leadership and clinical support, adequate continuous professional education, career mobility and career hierarchies, flexibility, planning and placement, professional admiration, provision of safety for work related diseases, and better salaries [6].

Studies have shown that employees' satisfaction with their QWL would improve performance, reduce absence on a job, reduce professional draining, reduce work related injuries, and increase job pleasure and satisfaction with most aspects of life in general [3, 7]. Another study indicated that employees who were gratified with their QWL work with greater interest, are more devoted to the organization, and are more productive [8].

Findings from a study conducted in Saudi Arabia indicated that 52.4% of nurses, particularly primary health care unit (PHCU) nurses, are dissatisfied with their quality of work life [9]. This was as high as 70.8% among Iranian nurses [10].

Earlier studies revealed that poor QWL was related to lack of independence to make patient care decisions, increased workload, role conflicts, lack of opportunities for career advancement, low salary, lack of professional autonomy, lack of stakeholders support and insufficient welfare services, attitude of society towards nursing, higher level of education, and longer professional experience which were factors that adversely affected the quality of work life [11–16].

Also, the result of previous studies implied that major influencing factors for dissatisfaction with QWL among nurses were unsuitable work hours, inability to balance work with family needs, insufficiency of breaks time, poor employment, delay in promotion, and insufficient hospital sponsored training [17–19].

Studies from Iran and Taiwan showed that nurses working in outpatient case teams revealed better quality of work life than nurses working in other departments. Nurses working in inpatient departments tend to require shift work, direct patient contact for care, and high time burden, work overload, and environmental conditions thus resulting in lower QWL [20, 21].

In terms of work environment, results from the University of Western Ontario found that nurses were dissatisfied with the security department with resultant concerns about safety in the work place. It also found that pays and benefits play a crucial role in determining employees' QWL satisfaction. In addition, unfavorable work environments can lead to low performance and poor interpersonal relationships among nurses that lead them to leave the facility or even the profession itself [22].

Notably, enhancing the QWL is an all-inclusive course of a process to improve the quality of life of workforces in the facilities and is crucial in any organization to recruit and hold its staffs. A high quality of work life (QWL) is a crucial issue for health care facilities to have a qualified, dedicated, and inspired employee. Service provision in the health care facilities depends on the capacity and capabilities of their man power [23, 24]. Among different specialties in health care settings, nurses have a major share among other health care providers [25], and enhancing their work life quality has become a critical issue in health care settings [26, 27].

Health care institutions in Ethiopia, as in the rest of the world, are experiencing problems with the provision of quality care in health care settings. According to the study conducted on professionalism in Mekelle public hospital, the quality of care received by patients is largely associated with the quality of work life practiced by health care providers. The quality of nursing care in health care settings is only achievable if nurses experience a better quality of their work life because nurses have a pivotal role in the delivery of care at all levels of health care facilities [28].

It is widely believed that the main duty of any health facility manager is to explore and promote the quality of employees' work life by continuously evaluating their work setup and identifying likely failings [29].

In recent times, there has been a shared assertion that nurses' attitude towards delivering work is poor in health care facilities. However, if nurses are thoroughly observed at work, we may ask these questions: are nurses really gratified with their quality of work life? What are the possible predictors that may be linked with their work life quality? For this, there is a paucity of studies in Ethiopia which explain the QWL among nurses in public health care settings. Hence, we aimed to find out the level of quality of work life and its determinants among nurses working in public health facilities in Hawassa, South Ethiopia.

## 2. Methods

### 2.1. Study Area and Study Design

Facility based cross-sectional study was conducted from March 10 to March 27, 2016, in nine health centers (HCs) and two hospitals of Hawassa town, South Ethiopia. Hawassa is located on the eastern shore of Lake Hawassa 275 km south of Addis Ababa with an area of 50 square kilometers. The town has a projected population of 328,834 for 2015/16 of whom 167,705 are females. The town has one referral hospital, one general hospital, and nine health centers which are all government owned facilities. The composition of health professionals in these facilities includes 710 nurses, 137 physicians, and other health care providers.

### 2.2. Participants

The source population included all nurses who were working in Hawassa town public health facilities (government owned). A random sample of nurses working as full timers in nine health centers (HCs) and two hospitals of Hawassa town was included in the study. The random sampling was accomplished by using a sampling frame at each health care facility through lottery method. For this, pieces of papers are folded and mixed into a box; the samples were taken randomly from the box by choosing folded pieces of papers in a random manner without replacement.

The sample size was determined using the formula for sample size determination for estimation of a single population proportion assuming population proportion (p) of 50% for nurses who were dissatisfied with the quality of work life. This was preferred for the sample size determination due to lack of similar studies in Ethiopia. Other assumptions made during the sample size calculation were 5% marginal error (d) and confidence interval of 95%. Since the source population is 710 which is less than 10,000, using finite population correction formula and adding 10% nonresponse rate, the final sample was 274. Because of the nature of random sampling technique and resource and time issues, oversampling was not employed. The total sample was proportionally allocated based on the number of nurses in the study facilities. A sampling of nurses from the two hospitals and nine health centers was done using simple random sampling. A nurse was included in the study if he/she had a qualification of diploma and above with experience of more than 6 months in the profession at the time of the study.

### 2.3. Variables

The rationale for the background variables was review of literatures at global and national as well as regional levels and the variables were selected, adapting from different reviews having a conceptual framework.

The dependent variable was the level of quality of work life and the independent variables included background variables (age, sex, marital status, educational status, monthly income, work experience, working unit, dependent family, and working institution) and work environment.

### 2.4. Instruments

Data were collected using pretested Likert scale type self-administered questionnaires. Trained data collectors were recruited for each health care facility. The authors carried out an extensive supervision during the data collection on daily basis. The instruments were adapted from Brooks B, quality of nursing work life which was validated globally in different countries and reconsidered for its reliability after carrying out pretest on 5% of the sample participants. The instrument choice was because of the proximity to the study participants in measuring the outcome variable. The tool had three parts.

The first part was about background characteristics of participants including age, sex, marital status, educational status, type of health facility, monthly income, work experience, working unit, and presence of dependent family. The second part was regarding the quality of work life (QWL) measured using a questionnaire having a total of 32 items with four dimensions. These dimensions were work life/home life dimension measured with 4 items, the work design dimension measured with 7 items, the work context dimension measured with 17 items, and the work world dimension measured with 4 items. The tool was a 5-point Likert scale with 1 denoting strongly disagree through 5 denoting strongly agree. The interitem consistency of the scale as measured by the Cronbach's alpha value was 0.86 [30].

The third part consisted of work environment measurement scale which had a total of 11 items adapted from the previous study [31]. The items were rated on a 5-point Likert scale with 1 denoting strongly disagree through 5 denoting strongly agree. The scale demonstrated high interitem consistency with a Cronbach's alpha of 0.83.

*Quality of Work Life (QWL)*. It was measured by the Brooks quality of work life questionnaire which has a total of 32 items having 5-point Likert scale with 1 denoting strongly disagree to 5 denoting strongly agree. The minimum possible score is 32 and a maximum possible score is 160 and the higher the tertile the better the quality of work life. It was categorized as low, moderate, and high using a terrible classification of the quality of the work life total score. The same was applied for the subdimensions of quality of work life. 

*Work Environment*. It was measured with a total of 11 items adapted from the previous study. The minimum possible score is 11 and the maximum possible score is 55, rating from a 5-point Likert scale with 1 denoting strongly disagree to 5 denoting strongly agree. It was ranked as unfavorable, somewhat favorable, and favorable based on the terrible score.

### 2.5. Data Processing and Analysis

Data were checked for completeness every day and the responses in the completed questionnaire were coded and entered into Epi-Data version 3.1 and exported to SPSS version 20 for analysis. Descriptive statistics were generated to summarize the data. Multinomial logistic regression was performed to identify significant predictors of quality of work life. Three models were developed for the analysis to examine the effect of different categories of independent variables on the dependent variable. The first model assessed the effect of sociodemographic variables while in the second model the effect of work environment was examined. From the above two models, independent variables which had statistically significant association with the dependent variable (*p* < 0.05) were entered into the final multinomial logistic regression model based on a likelihood ratio test. An adjusted odds ratio (AOR) at 95% confidence interval (CI) was considered to declare an independent effect of explanatory variables on the outcome variable and corresponding *p* value set at less than 0.05.

## 3. Results

### 3.1. Sociodemographic Characteristics of Study Participants

Out of the 274 proposed nurses, 253 completed the questionnaire making the response rate of 92.33%. The response rate was good because of the topic's implication in the real life of nurses serving in the public health facilities of the study area at large. The mean age of the participants was 27.43 (±6.43) years ranging from 21 to 50 years. One hundred thirty-five (53.4%) of the participants were single and more than half of the participants were females. The mean (±SD) years of experience of the respondents were 4.32 (±3.32) ranging from 1 to 21 years of service. About 60% of the participants work in hospitals and more than one-third (40.7%) of the nurses work in the inpatient unit of the facilities. In the study 153 (60.5%) were diploma holders. The mean (±SD) gross monthly income for the respondents was 143 (±55.77) USD ranging from 87 to 292 USD and for nearly two-thirds (59.7%) of the nurses the gross monthly salary was below 143 USD (Table 1).

### 3.2. Level of Quality of Work Life

**Figure 1:**
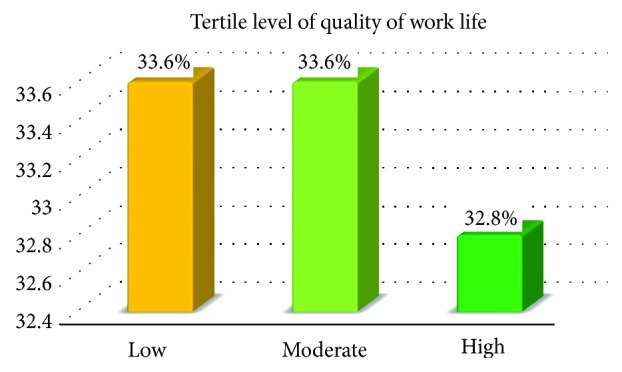
Tertile classification of quality of work life among nurses working in public health facilities of Hawassa town, March 10–27, 2016.

The actual range for the QWL score of the study participants was 50 to 129 with a mean (±SD) of 92.23 (±15.85). This finding implied that 67.2% of the respondents were dissatisfied with their quality of work life. Based on tertile classification using rank cases 33.6% of the nurses reported that they felt a low and moderate level of quality of work life while the remaining 32.8% rated experiencing a relatively high level of quality of work life (Figure 1).

### 3.3. Quality of Work Life among Nurses Based on Dimensions

#### 3.3.1. Work Life/Home Life Dimension

The actual range score of the current study was 4 to 18 with a mean (±SD) of 10.46 (±2.65). The majority of the respondents, 217 (88.5%), were not able to balance work life with their family desires. Half of the respondents, 128 (50.6%), agreed that they are not happy with working hours which do not suit their daily life and 180 (71.1%) stated that they experience fatigue after work. Two-thirds (66.4%) of the respondents felt that the policy of their health care organizations for vacation is not appropriate either for themselves or for their families.

#### 3.3.2. Work Design Dimension

The actual score for this dimension in the current study ranged from 11 to 32 with a mean (±SD) of 22.54 (±4.26). Nearly two-thirds (62.1%) of the respondents reported that their workload is heavy including accomplishment of nonnursing tasks and 158 (62.5%) agreed that they do not have an independence to make decisions to provide a client or patient care. However, 146 (57.7%) of respondents reported that there are enough nurses in their health care facilities.

#### 3.3.3. Work Context Dimension

The actual range score of the current study was 17 to 85 with a mean (±SD) of 49.52 (±9.97). Management and supervision issues were of concern. One hundred fifty-two (60.1%) of the respondents reported that they do not obtain both satisfactory supportive supervision and feedback from their nurse manager/supervisor and only 101 (39.9%) felt recognized for their accomplishments. Regarding participation, 163 (64.4%) of the respondents stated that they have no chance of participating in decision-making courses. Additionally, two-thirds (69.2%) of the respondents stated that nursing strategies and processes are not supportive enough and only 106 (41.9%) of the nurses felt respected by the respective management bodies.

In terms of professional development opportunities, only 85 (33.6%) of the respondent nurses agreed that it is important to have the opportunity to further their nursing education without leaving their current job. More importantly, 219 (86.6%) of the nurses revealed that they do not obtain support to join continuing education and training programs. Moreover, 188 (74.3%) of the participants reported that their organizations do not provide adequate opportunities for career advancement.

This study showed that nurses were remarkably satisfied with factors related to their coworkers except for physicians. One hundred seventy-nine (70.8%) of the nurses reported that there is teamwork in their health facility and 207 (81.9%) revealed that they have good relationships with their coworkers. Around three-fourths (70.8%) of respondents revealed that they have better communication with other staffs. However, only 96 (38%) of the nurses agreed that they have good communication with physicians. Even more disturbing was the fact that only 66 (26.1%) of the nurses felt respected by physicians.

Despite expressing that they were not satisfied with the quality of their work life, more than half of the respondents, 159 (62.9%), expressed a sense of belongingness in their health care settings.

#### 3.3.4. Work World Dimension

The actual range score of the current study was 4 to 18 with a mean (±SD) of 10.46 (±2.65). About 214 (80.7%) of the nurses in this study did not think the society has an accurate image of nurses. However, about three-quarters (75.9%) of the nurses believed that nursing work has a positive impact on the lives of others, indicating excellent attitudes towards their profession as well as a special sense of self-image. Salary was also an essential factor that contributes to disappointment among nurses working in public health facilities. The majority (93.7%) of the respondents reported that their payment is not adequate considering the nature of duties they are accomplishing and only 42 (20.5%) of the respondents believed that their jobs are secured (Table 2).

### 3.4. Work Environment Score

The actual mean (±SD) of work environment score was 23.99 (±7.46). In the current study, the minimum reported score was 11 and the maximum was 46 from a total score of 55. Based on tertile classification only 35% of the nurses rated experiencing a relatively favorable work environment.

### 3.5. Background Characteristics as Predictors of Quality of Work Life

In this model, educational status, monthly income, and unit of work were found to be significantly associated with nurses' quality of work life score (*p* < 0.05). With a high quality of work life as a reference, diploma holders were 4.75 times more likely to experience a low quality of work life than those who had a bachelor degree (AOR = 4.750, 95% CI = 1.349–16.745). Conversely, respondents who had diploma were 6.198 times less likely to experience a moderate quality of work life than those who had a bachelor degree (AOR = 6.198, 95% CI = 1.793–21.427). The pseudo-*R*-square value showed that this model explained 23.1% of the variation (Table 3).

### 3.6. Work Environment and Quality of Work Life among Nurses

The likelihood ratio test in Table 4  shows the relation between QWL and work environment. Compared to those who experienced a high quality of work life, respondents who perceived unfavorable work environment were 10 times more likely to experience a low quality of work life than those who perceived favorable work environment (AOR = 10.328, 95% CI = 4.408–24.202). On the other hand, compared to those who experienced a high quality of work life, respondents who perceived somewhat favorable work environment were 9 times more likely to feel a low quality of work life than those who perceived favorable work environment (AOR = 9.241, 95% CI = 3.916–21.806). The pseudo-*R*-square value showed that this model explained 21% of the variation (Table 4).

### 3.7. Independent Predictors of Quality of Work Life among Nurses

The last model was developed by entering all the variables shown to have a statistically significant association (*p* < 0.05) with nurses' quality of work life in the earlier two models. In this model, the pseudo-*R*-square implied that the model explained about 38.9% of the variance and it fitted the data adequately (*p* > 0.937).

Educational status and work environment were found to be significant predictors (*p* < 0.05) of both low and moderate quality of work life among nurses. However, monthly income was a significant predictor of low quality of work life but not a moderate quality of work life among nurses. Unit of work was significantly associated with a moderate level of nurses' quality of work life.

With a high quality of work life as a reference, nurses with a monthly income less than 3145 Eth Birr were 12 times more likely to experience a low quality of work life compared to those earning greater than 5583 Eth Birr (AOR = 12.00, 95% CI = 1.463–18.423) (Table 5(a)).

With a high quality of work life as a reference, nurses working in the outpatient unit were 3.143 times more likely to experience a moderate quality of work life compared to those who are working in other units (AOR = 3.143, 95% CI = 1.082–9.132) (Table 5(b)).

## 4. Discussion

This study was carried out with the aim of determining the level of quality of work life (QWL) and associated factors among nurses. This is important because health care facilities need qualified nurses and want to understand how to retain and develop competent staff compositions. Moreover, efficient QWL programs can improve the morale of employees and organizational effectiveness and improve the quality of nursing care [13]. This study implied that 67.2% of the respondents were dissatisfied with their quality of work life. Similarly, earlier studies from Saudi Arabia, Iran, and Nigeria reported a dissatisfaction rate of 52.4% to 68.8% [9, 10, 14, 18].

This study revealed that the quality of work life among nurses was influenced by educational status, monthly income, work unit, and the work environment. More specifically, respondents who had diploma were more prone to experience a low quality of work life (AOR = 4.750). This study also found that the QWL of nurses with lower educational status was lower than nurses with higher educational status. This finding was consistent with the result of a study conducted in Tamale teaching hospital in Ghana [15]. However, another study from Iran showed that QWL of nurses with a lower level of education was better than nurses with higher educational status [14]. The very low salary compounded with high workload encountered by junior nurses in Ethiopia might explain their experience of low QWL.

In support of this, the current study demonstrated a significant association between QWL of nurses and their monthly income (AOR = 12.000). Only 11 (4.3%) of the nurses reported gross monthly above 5583 Ethiopian Birr (ETB) or 254 USD which is the initial salary for master's degree holders in public health facilities. The majority (59.7%) of the respondents reported that their salary is not adequate considering the nature of duties they are accomplishing in the health facilities. These findings are in line with the results reported by studies conducted in Iran and Saudi Arabia [9, 24]. In another study, Lewis and colleagues concluded that pays and benefits play a crucial role in determining employees' satisfaction with QWL [22].

The current study also found that the work unit of the respondents had statistically significant association with quality of work life among nurses. Nurses who were working in outpatient departments were more likely to experience a moderate level of quality of work life (AOR = 3.143). Similarly, a study conducted in Taiwan revealed that nurses working in outpatient departments exhibited a better quality of life than nurses working in other units [21]. This could relate to the fact that units other than the outpatient departments usually require engagement in night and weekend shift duty, direct patient care, and work overload which could result in lower quality of life.

The results also showed that the work environment of the health care facilities was strongly significantly associated with quality of work life among nurses. Nurses who perceived unfavorable work environment reported a low quality of work life (AOR = 10.328). Similarly, previous studies conducted in Iran among nurses highlighted concerns about the safety of the work environment as a major factor in nurses' dissatisfaction with their workplaces [12, 20].

The result in this study has indicated that age, sex, marital status, years of experience, and type of institution had no significant relationship with QWL (*p* > 0.05). On the contrary, a study conducted in Iran revealed that there is a close relation between age and QWL [10]. In a similar study in Nigeria, a significant relationship was found between work experience and QWL [18]. A study conducted in Egypt indicated that the perception of QWL among nurses was significantly higher with advanced age and longtime service [19].

Moreover, the promotion opportunities and professional growth had an influential impact on the QWL of nurses. When the nurses feel dissatisfied with their future promotion and career development, their quality of work life will be affected negatively. A study in Saudi Arabia reported the impact of professional development opportunities such as the promotion system, access to degree programs, and continuing education on the QWL of nurses [9].

A study from Nigeria showed that the nurses felt that lack of opportunities for educational advancement and hospital sponsored training and inability to influence decisions which are issues that affect the QWL [18]. In this study, more than half (62.5%) of the respondents agreed that they do not have an autonomy to make client or patient care decisions in their facilities. Similarly, in a study from Ghana, the majority (76.52%) of the nurses expressed the view that they were not given autonomy often to decide how jobs should be performed [15].

Interpretation of the comparisons we have made above should be made being mindful of the health institutional setup and health policy differences between the study area and the countries in which the cited studies were conducted.

### 4.1. Practical Implications

In the 21st century, we are striving to deliver a quality of care, improve patient satisfaction, change the public image, and as a whole achieve population health improvement. This will have also a great impact to enhance productivity and attain organizational goals easily. But, we cannot achieve all these goals by having nurses with a low level of quality of work life including the majority of the health care team in any health care setting.

## 5. Conclusion

We found that more than six in ten of the nurses included in the study were dissatisfied with their quality of work life. The finding of this study adds a small but essential piece to the puzzle of how to maintain the quality of work life among nurses in the health care facilities in Ethiopia. The study found that independent predictors of quality of work life among the study population were educational status, monthly income, working unit, and work environment.

The findings in this study and studies reported from elsewhere pinpoint that perception of nurses about the quality of their work life can be modified if health care managers are considerate of the key issues surrounding QWL. We recommend that the incentive and remuneration packages, workplace arrangements, and opportunities for further education and career development should be reexamined to satisfy the concerns of the nurses in the study facilities.

## Figures and Tables

**Table 1:** Background characteristics of nurses working in Hawassa town public health facilities, March 10–27, 2016 (*n* = 253).

Participant nurses characteristics	Frequency	Percentage
Sex		
Male	121	47.8
Female	132	52.2
Age		
20–24	46	18.2
25–29	168	66.4
30–34	29	11.5
≥35	10	4.0
Marital status		
Married	118	46.6
Single	135	53.4
Educational status		
Diploma	153	60.5
Bachelor degree	99	39.1
Master's degree	1	0.4
Work experience		
Up to 2 years	77	30.4
2–5 years	115	45.5
6–10 years	47	18.6
≥11 years	14	5.5
Monthly income (in ETB)		
<3145	151	59.7
3145–5582	91	36.0
≥5583	11	4.3
Institution		
Health center	105	41.5
Hospital	148	58.5
Unit of work		
Outpatient	65	25.7
Inpatient	103	40.7
Emergency	54	21.3
Delivery	31	12.3

**Table 2:** Description of the quality of work life scale items among nurses working in Hawassa town public health facilities, March 10–27, 2016 (*n* = 253).

Quality of work life scale items (*α* = 0.86)	Dissatisfied		Satisfied	
	Number	%	Number	%
*Work/home life dimension items*				
I have enough energy left after work.	180	71.1	73	28.9
I am able to balance work with my family needs.	217	85.8	36	14.2
My organization's policy for vacations is appropriate for me and for my family.	168	66.4	85	33.6
The system of working hours in the healthcare facility negatively affects my life.	128	50.6	125	49.6
*Work design dimension items*				
I feel comfortable and satisfied with my job.	168	66.4	85	33.6
My workload is too heavy.	157	62.1	96	37.9
I have the autonomy to make client/patient care decisions.	158	62.5	95	37.5
I perform many non-nursing tasks.	134	53.0	119	47.0
There are enough nurses in my work setting.	107	42.3	146	57.7
I have enough time to do my job well.	97	38.3	156	61.7
I am able to provide good quality client/patient care.	57	22.5	196	77.5
*Work context dimension items*				
I am recognized for my accomplishments by my nurse manager/supervisor.	152	60.1	101	39.9
I am able to participate in decisions made by my nurse manager/supervisor.	163	64.4	90	35.6
I am able to communicate well with my nurse manager/supervisor.	74	29.2	179	70.8
I receive feedback on my performance from my nurse manager/supervisor.	152	60.1	101	39.9
Upper-level management has respect for nursing.	147	58.1	106	41.9
Existing nursing policies and procedures are good enough to facilitate my work.	175	69.2	78	30.8
I feel respected by physicians in my work setting.	187	73.9	66	26.1
I communicate well with the physicians in my work setting.	157	62.1	96	37.9
Friendships/relationships with my co-workers are acceptable.	46	18.2	207	81.8
I feel like there is teamwork in my work setting.	74	29.2	179	70.8
My work setting provides career advancement opportunities.	188	74.3	65	25.7
I believe that it is important to have the opportunity to further my nursing education without leaving the current job.	168	66.4	85	33.6
I receive support to attend continuing education and training programs.	219	86.6	34	13.4
I have adequate client/patient care supplies and equipment.	152	60.1	101	39.9
It is important to have a designated private break area for the nursing staff.	121	47.8	132	52.2
I feel safe from personal harm (physical, emotional or verbal) at work.	191	75.5	62	24.5
I feel a sense of belonging in my workplace.	94	37.2	159	62.8
*Work world dimension items*				
My work impacts the lives of patients, families and the community.	85	33.6	168	66.4
I believe that, in general, society has an accurate image of nurses.	204	80.6	49	19.4
I feel quite secured about my job.	201	79.4	52	20.6
My salary is adequate for my job, given the current job market conditions and workload.	237	93.7	16	6.3

**Table 3:** Background characteristics as predictors of quality of work life among nurses working in public health facilities of Hawassa town, March 10–27, 2016 (*n* = 253).

Explanatory variables	Quality of work life (predicted)			
	Low		Moderate	
	*p*	AOR (CI)	*p*	AOR (CI)
Sex				
Male	0.087	1.890 (0.911–3.921)	0.981	1.009 (0.490–2.077)
Female^†^	1	1	1	1
Age				
20–24	0.578	0.527 (0.055–5.036)	0.137	0.194 (0.022–1.687)
25–29	0.755	0.709 (0.082–6.145)	0.434	0.445 (0.059–3.378)
30–34	0.335	0.312 (0.029–3.338)	0.656	0.608 (0.068 = –5.438)
≥35^†^	1	1	1	1
Marital status				
Married	0.947	0.974 (0.449–2.112)	0.328	0.681 (0.316–1.470)
Single^†^	1	1	1	1
Educational status				
Diploma	0.015	4.750 (1.349–16.745)	0.004	6.198 (1.793–21.427)
Bachelor degree^†^	1	1	1	1
Work experience				
Up to 2 years	0.967	1.048 (0.108–10.200)	0.880	1.203 (0.110–13.199)
2–5 years	0.778	1.376 (0.150–12.663)	0.635	1.760 (0.170–18.215)
6–10 years	0.917	0.890 (0.097–8.124)	0.832	0.777 (0.076–7.989)
≥11 years^†^	1	1	1	1
Monthly income				
<3145	0.003	0.012 (0.001–0.225)	0.274	0.146 (0.005–4.604)
3145–5583	0.019	0.051 (0.004–0.615)	0.626	0.464 (0.021–10.179)
≥5583^†^	1	1	1	1
Institution				
Health center	0.167	0.590 (0.280–1.247)	0.153	0.587 (0.282–1.220)
Hospital^†^	1	1	1	1
Unit of work				
Outpatient	0.076	0.349 (0.109–1.118)	0.035	3.143 (1.082–9.132)
Inpatient	0.270	0.527 (0.169–1.647)	0.719	0.817 (0.272–2.456)
Emergency	0.633	1.348 (0.396–4.591)	0.957	0.967 (0.284–3.286)
Delivery^†^	1	1	1	1

Reference category for outcome variables: high. AOR: adjusted odds ratio. ^†^Reference category for explanatory variables.

**Table 4:** Work environment and quality of work life among nurses working in public health facilities of Hawassa town, March 10–27, 2016 (*n* = 253).

Explanatory variables with response options	Level of quality of work life (predicted)			
	Low		Moderate	
	*p*	AOR (CI)	*p*	AOR (CI)
Work environment				
Unfavorable	0.001	10.328 (4.408–24.202)	0.001	4.206 (1.861–9.508)
Somewhat favorable	0.001	9.241 (3.916–21.806)	0.001	6.562 (3.005–14.329)
Favorable^†^	1	1	1	1

Reference category for outcome variables: high. AOR: adjusted odds ratio. ^†^Reference category for explanatory variables.

**Table:** (a) Predictors of low quality of work life among nurses working in public health facilities of Hawassa town, March 10–27, 2016 (*n* = 253)

Predictors	Parameter estimates			
	*B*	df	*p*	AOR (CI)
Sex				
Male	0.636	1	0.087	1.890 (0.911–3.921)
Female^†^		0	1	1
Age				
20–24	−0.641	1	0.578	0.527 (0.055–5.036)
25–29	−0.343	1	0.755	0.709 (0.082–6.145)
30–34	−1.165	1	0.335	0.312 (0.029–3.338)
≥35^†^		0	1	1
Marital status				
Married	−0.026	1	0.947	0.974 (0.449–2.112)
Single^†^	1	0	1	1
Educational status				
Diploma	1.558	1	0.015	4.750 (1.349–16.745)
Bachelor degree^†^	1	0	1	1
Work experience				
Up to 2 years	0.047	1	0.967	1.048 (0.108–10.200)
2–5 years	0.319	1	0.778	1.376 (0.150–12.663)
6–10 years	−0.117	1	0.917	0.890 (0.097–8.124)
≥11 years^†^	1	0	1	1
Monthly income				
<3145	2.485	1	0.021	12.000 (1.463–18.423)
3145–5583	2.003	1	0.065	7.412 (0.885–12.099)
≥5583^†^	1	0	1	1
Institution				
Health center	−0.527	1	0.167	0.590 (0.280–1.247)
Hospital^†^	1	0	1	1
Unit of work				
Outpatient	−1.054	1	0.076	0.349 (0.109–1.118)
Inpatient	−0.641	1	0.270	0.527 (0.169–1.647)
Emergency	0.299	1	0.633	1.348 (0.396–4.591)
Delivery^†^	1	0	1	1
Work environment				
Unfavorable	2.335	1	0.001	10.328 (4.408–24.202)
Somewhat favorable	2.224	1	0.001	9.241 (3.916–21.806)
Favorable^†^	1	0	1	1

The reference category for the outcome variable: high. AOR, adjusted odds ratio; *B*, estimated regression coefficient; df, degrees of freedom. ^†^Reference category for the explanatory variables.

**Table:** (b) Predictors of moderate quality of work life among nurses working in public health facilities of Hawassa town, March 10–27, 2016 (*n* = 253)

Predictors	Parameter estimates			
	*B*	df	*p*	AOR (CI)
Sex				
Male	0.009	1	0.981	1.009 (0.490–2.077)
Female^†^	1	0	1	1
Age				
20–24	−1.639	1	0.137	0.194 (0.022–1.687)
25–29	−0.809	1	0.434	0.445 (0.059–3.378)
30–34	−0.498	1	0.656	0.608 (0.068 = –5.438)
≥35^†^		0	1	1
Marital status				
Married	−0.384	1	0.328	0.681 (0.316–1.470)
Single^†^	1	0	1	1
Educational status				
Diploma	1.824	1	0.004	6.198 (1.793–21.427)
Bachelor degree^†^	1	0	1	1
Work experience				
Up to 2 years	0.185	1	0.880	1.203 (0.110–13.199)
2–5 years	0.565	1	0.635	1.760 (0.170–18.215)
6–10 years	−0.253	1	0.832	0.777 (0.076–7.989)
≥11 years^+^	1	0	1	1
Monthly income				
<3145	−1.926	1	0.274	0.146 (0.005–4.604)
3145–5583	−0.768	1	0.626	0.464 (0.021–10.179)
≥5583^†^		0	1	1
Institution				
Health center	−0.533	1	0.153	0.587 (0.282–1.220)
Hospital^†^		0	1	1
Unit of work				
Outpatient	1.145	1	0.035	3.143 (1.082–9.132)
Inpatient	−0.202	1	0.719	0.817 (0.272–2.456)
Emergency	−0.034	1	0.957	0.967 (0.284–3.286)
Delivery^†^		0	1	1
Work environment				
Unfavorable	1.437	1	0.001	4.206 (1.861–9.508)
Somewhat favorable	1.881	1	0.001	6.562 (3.005–14.329)
Favorable^†^	1	0	1	1

The reference category for the outcome variable: high. AOR, adjusted odds ratio; *B*, estimated regression coefficient; df, degrees of freedom. ^†^Reference category for the explanatory variables.

## Abbreviations

AOD: Adjusted odds ratio
CI: Confidence interval
ETB: Ethiopian Birr
IRB: Institutional Review Board
HCs: Health centers
PHCU: Primary health care unit
QWL: Quality of work life
SPSS: Statistical Package for Social Sciences.

## Additional Points

*Availability of Data and Materials*. The spreadsheet data supporting the findings of this work is available at the hands of the corresponding author.
